# Regulation and splicing of scavenger receptor class B type I in human macrophages and atherosclerotic plaques

**DOI:** 10.1186/1471-2261-5-25

**Published:** 2005-08-25

**Authors:** Per-Arne Svensson, Mikael CO Englund, Magnus SC Snäckestrand, Daniel A Hägg, Bertil G Ohlsson, Veronika Stemme, Lillemor Mattsson-Hulten, Dag S Thelle, Björn Fagerberg, Olov Wiklund, Lena MS Carlsson, Björn Carlsson

**Affiliations:** 1 Research Centre for Endocrinology & Metabolism, Department of Internal Medicine, The Sahlgrenska Academy, Göteborg University, S-413 45 Göteborg, Sweden; 2 The Wallenberg Laboratory for Cardiovascular Research, The Sahlgrenska Academy, Göteborg University, S-413 45 Göteborg, Sweden; 3 Cardiovascular Research Unit, Center for Molecular Medicine, Karolinska Institute, Stockholm, Sweden; 4 Department of Medicine, Cardiovascular Institute, The Sahlgrenska Academy, Göteborg University, Göteborg, Sweden; 5 Department of Body Composition and Metabolism, The Sahlgrenska Academy, Göteborg University, Göteborg, Sweden

## Abstract

**Background:**

The protective role of high-density lipoprotein (HDL) in the cardiovascular system is related to its role in the reverse transport of cholesterol from the arterial wall to the liver for subsequent excretion via the bile. Scavenger receptor class B type I (SR-BI) binds HDL and mediates selective uptake of cholesterol ester and cellular efflux of cholesterol to HDL. The role of SR-BI in atherosclerosis has been well established in murine models but it remains unclear whether SR-BI plays an equally important role in atherosclerosis in humans. The aim of this study was to investigate the expression of SR-BI and its isoforms in human macrophages and atherosclerotic plaques.

**Methods:**

The effect of hypoxia and minimally modified low-density lipoprotein (mmLDL), two proatherogenic stimuli, on SR-BI expression was studied in human monocyte-derived macrophages from healthy subjects using real-time PCR. In addition, SR-BI expression was determined in macrophages obtained from subjects with atherosclerosis (n = 15) and healthy controls (n = 15). Expression of SR-BI isoforms was characterized in human atherosclerotic plaques and macrophages using RT-PCR and DNA sequencing.

**Results:**

SR-BI expression was decreased in macrophages after hypoxia (p < 0.005). In contrast, SR-BI expression was increased by exposure to mmLDL (p < 0.05). There was no difference in SR-BI expression in macrophages from patients with atherosclerosis compared to controls. In both groups, SR-BI expression was increased by exposure to mmLDL (p < 0.05). Transcripts corresponding to SR-BI and SR-BII were detected in macrophages. In addition, a third isoform, referred to as SR-BIII, was discovered. All three isoforms were also expressed in human atherosclerotic plaque. Compared to the other isoforms, the novel SR-BIII isoform was predicted to have a unique intracellular C-terminal domain containing 53 amino acids.

**Conclusion:**

We conclude that SR-BI is regulated by proatherogenic stimuli in humans. However, we found no differences between subjects with atherosclerosis and healthy controls. This indicates that altered SR-BI expression is not a common cause of atherosclerosis. In addition, we identified SR-BIII as a novel isoform expressed in human macrophages and in human atherosclerotic plaques.

## Background

The presence of lipid-loaded macrophages (foam cells) in the vascular wall is one of the hallmarks of atherosclerosis. High levels of low-density lipoprotein (LDL) cholesterol increase the risk of atherosclerosis and the lipids accumulated in the macrophages are derived mainly from modified forms of LDL. Oxidation of LDL is believed to be the major modification of LDL cholesterol *in vivo *[1]. As the atherosclerotic plaque increases in size due to accumulation of lipid and fibrous elements, local supply of oxygen and nutrients may become insufficient. In advanced atherosclerotic plaques, zones of hypoxia have been identified [2,3].

The level of high-density lipoprotein (HDL) cholesterol is inversely related to the risk of developing atherosclerosis. A proposed mechanism for the athero-protective role of HDL is the transfer of cholesterol from the arterial wall via the liver to the bile, a process known as reverse cholesterol transport [4]. Scavenger receptor class B type I (SR-BI; also known as CLA-1) has been shown to mediate selective uptake of cholesterol esters from HDL particles into cells [5]. Moreover, SR-BI mediates cholesterol efflux *in vitro *[6,7] and has been suggested to contribute to cellular cholesterol efflux also *in vivo*. Therefore, SR-BI may be of importance also for the first step in reverse cholesterol transport, i.e. the removal of cholesterol from the arterial wall [4].

The role of SR-BI in HDL metabolism and atherosclerosis has been well established in murine models. Experiments in rodents have shown that SR-BI is expressed in tissues with selective cholesterol uptake, *e.g*. liver, adrenals, ovaries and testes [5,8]. Hepatic overexpression of SR-BI in transgenic mice decreases the development of atherosclerosis in cholesterol-fed LDL receptor knockout (KO) mice [9]. Recent studies have demonstrated that lack of SR-BI expression in macrophages increases atherosclerotic lesion formation in mice [10]. The link between human SR-BI and atherosclerosis in man is less well established. Human SR-BI mediates selective cholesterol uptake into cells and displays a tissue distribution similar to that in rodents [11,12]. SR-BI expression has been detected in human atherosclerotic plaques [13] and variants of the human SR-BI gene have been associated to lipid abnormalities [14,15]. Recently, a variant of the human SR-BI promoter, that displays decreased expression, has been associated to increased plasma HDL cholesterol levels [16]. Therefore, decreased macrophage expression of human SR-BI may be a mechanism that promotes the development of atherosclerosis.

The aim of this study was to investigate the expression of SR-BI in human macrophages and atherosclerotic plaques.

## Methods

### Subjects and samples

Patients diagnosed with myocardial infarction or unstable angina pectoris were identified via the INTERGENE study, which is a population study recruiting subjects from Västra Götaland, Sweden. For details on the sampling and procedures in the INTERGENE study, see Berg *et al *[17]. First-degree relative (siblings, children or parents) of the patients were screened by ultra-sound examination (Sequoia ultra sound scanner) of the intima thickness of the carotid and femoral arteries to determine the extent of atherosclerosis. Fifteen subjects with sub-clinical atherosclerosis were recruited to this study (the macrophage INTERGENE study). The inclusion criteria for the atherosclerotic group were at least one atherosclerotic plaque in the carotid or femoral arteries and a verified family history of myocardial infarction or unstable angina pectoris, as documented in the INTERGENE study. Fifteen healthy control subjects matched by sex and age, which had no such family history, were also recruited from the INTERGENE population study. The exclusion criteria for both groups were, age <40 years, diabetes mellitus, clinical cardiovascular disease, smoking during the last 10 years, severe hypercholesterolemia, current infection (C-reactive protein >5 mg/L), and severe chronic disease. Additional exclusion criteria for the control group were hypertension.

Specimens from atherosclerotic carotid arteries were obtained from two females and two males undergoing carotid endarterectomy (n = 4).

The lipid analyses were performed at the Wallenberg Laboratory and the remaining biochemical analyses at the central laboratory at Sahlgrenska University Hospital. Triglyceride and cholesterol levels were analysed by fully enzymatic techniques [18,19]. LDL cholesterol was calculated as described by Friedewald et al. [20]. HDL was determined after precipitation of apolipoprotein (apo) B-containing lipoproteins with manganese chloride and dextran sulfate. The clinical and biochemical characteristics of the study groups (macrophage INTERGENE subjects) are presented in Table 1. The atherosclerotic subjects had higher total cholesterol levels and higher systolic blood pressure compared to the healthy control subjects.

**Table 1:** Clinical and biochemical characteristics of the study groups (The macrophage INTERGENE subjects).

**Characteristic**	**Healthy control subjects (n = 15)**	**Atherosclerotic subjects (n = 15)**
Age^1 ^(years)	57 (47–73)	58 (43–74)
Weight (Kg)	79 ± 13	80 ± 13
Systolic blood pressure (mmHg)	126 ± 14	144 ± 16*
Diastolic blood pressure (mmHg)	74 ± 8	78 ± 11
Serum Na (mmol/L)	140 ± 2	140 ± 2
Serum K (mmol/L)	4.10 ± 0.26	4.11 ± 0.27
Serum Ca (mmol/L)	2.36 ± 0.07	2.37 ± 0.07
Blood Hb (g/L)	142 ± 6	143 ± 9
Blood glucose (mmol/L)	5.1 ± 0.8	5.0 ± 0.7
Total cholesterol (mmol/L)	5.33 ± 0.64	5.99 ± 0.69*
LDL cholesterol (mmol/L)	3.23 ± 0.72	3.78 ± 0.75
HDL cholesterol (mmol/L)	1.56 ± 0.50	1.40 ± 0.49
Serum triglycerides (mmol/l)	1.37 ± 0.46	1.78 ± 1.07

Data are presented as means ± SD. ^1^mean and range. *P < 0.05 (Student's t-test).

The different studies in the project were approved by the ethics committees of Göteborg University and Karolinska Institute.

### Macrophage preparation and culture

Human mononuclear cells were isolated by Ficoll-Paque (Amersham Biosciences, Uppsala, Sweden) using buffy coats from the atherosclerotic subjects or healthy control subjects in the macrophage INTERGENE study or healthy volunteers. The mononuclear cell preparations were washed five times with PBS, pH 7.2, without calcium and magnesium, but containing 10 mM EDTA. The cell preparation was performed at room temperature. Mononuclear cells were resuspended and seeded at a density of 10^7 ^cells per each 100-mm plastic dish in a serum-free medium (Macrophage-SFM, GIBCO BRL; Grand Island, NY), supplemented with penicillin 100 U/ml and streptomycin 100 μg/ml. The mononuclear cells were allowed to adhere for 1 h. Non-adhered cells were eliminated with three washes with PBS. Adherent monocytes were cultured in Macrophage-SFM medium with antibiotics and supplemented with human granulocyte macrophage colony stimulating factor (GM-CSF), 70 U/ml (R&D Systems Europe Ltd., Abingdon, UK). The medium was discarded after 3d and the cells were washed once with PBS. The cells were then allowed to grow for another 3d in the same medium, but without GM-CSF and were then defined as monocyte-derived macrophages, and referred to as "macrophages" in the following text.

### Preparation of human LDL and cell treatment

Preparation of LDL was performed as previously described [21]. Minimally modified LDL (mmLDL) was generated by storing sterile native LDL in the dark at 4°C for three months (thiobarbituric acid-reactive substances (TBARS) = 5 nmol MDA/mg protein). Macrophages from healthy volunteers or from subjects in the macrophage INTERGENE study were incubated without (control) or with mmLDL (50 μg protein/ml) in Macrophage-SFM medium for 24 h. Macrophages from healthy volunteers were also incubated under normoxic (21% O_2_) or hypoxic (0% O_2_) conditions for 24 h as previously described [22]. Total RNA was isolated from the macrophages using RNeasy kit (Qiagen, Hilden, Germany).

### Real-time PCR analysis of SR-BI gene expression

Oligonucleotide primers and probes were designed with Primer Express 1.5 software (Applied Biosystems, Foster City, CA). Primers (forward: 5'-CCG CAC CTT CCA GTT CCA-3', reverse: 5'- ATG TTG GGC ATG ACG ATG TAG TC-3') and probe (5'-TCC AAG TCC CAC GGC TCG GAG A-3') were purchased from Applied Biosystems. These primers detects both SR-BI and the SR-BII isoform mRNA. The probes consisted of oligonucleotides that were labeled at the 5' end with the reporter dye FAM and at the 3' end with the quencher TAMRA. Reagents (TaqMan^® ^Reverse Transcriptase reagents and TaqMan^® ^Universal PCR Master mix, Applied Biosystems) and conditions were used according to the manufacturer's protocol. Briefly, cDNA was synthesized using reverse transcriptase (RT) from RNA samples. Each amplification reaction consisted of diluted cDNA (corresponding to 20 ng RNA), 300 nM of each primer, 200 nM TaqMan probe and TaqMan^® ^Universal PCR reaction mix. Amplification and detection of specific products were performed with the ABI Prism 7700 sequence detection system (Applied Biosystems) using default cycle parameters. Pre-developed assay reagents for human RPLP0 (large ribosomal protein) was obtained from Applied Biosystems and used as reference to normalize the expression levels between the samples. All standards and samples were analyzed in triplicates.

### Analysis of SR-BI isoforms by reverse transcriptase -PCR

**Figure 3:**
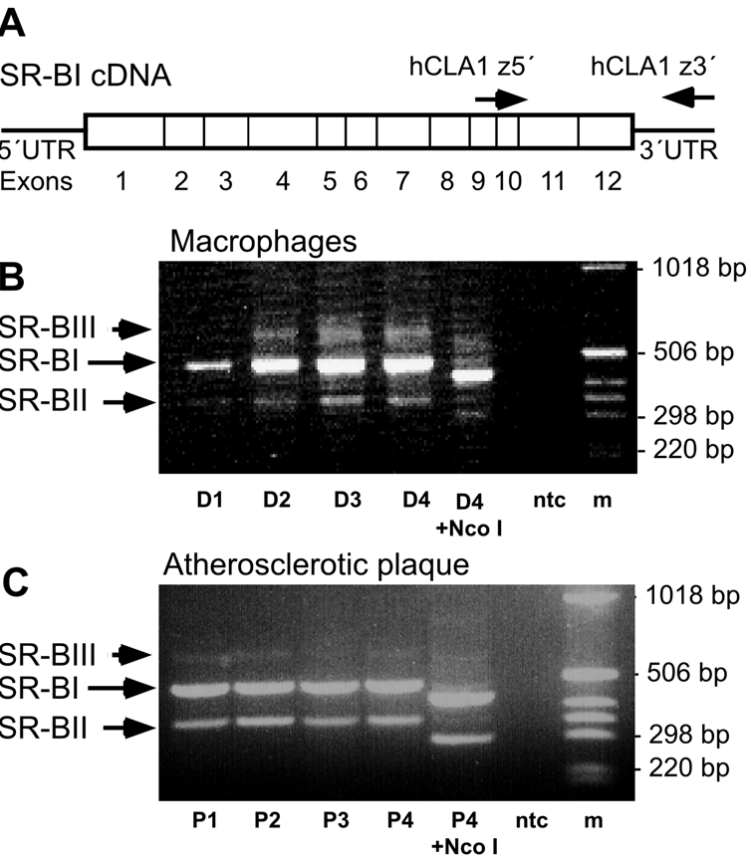
**Analysis of SR-BI expression in human macrophages and human carotid atherosclerotic plaque by RT-PCR**. Schematic illustration of the human SR-BI cDNA and the location of the primers used for PCR (A). Analysis of expression of SR-BI and SR-BI isoforms in RNA extracted from macrophages by RT-PCR (B). PCR products from four donors (D1–D4) and PCR products from donor 4 digested with Nco I (D4+Nco I), non-template control (ntc), 1 kb DNA-molecular marker (m). Analysis of expression of SR-BI and SR-BI isoforms in RNA extracted from atherosclerotic plaque tissue by RT-PCR (C). PCR products from atherosclerotic plaque tissue cDNA from four patients (P1–P4), PCR product from patient 4 digested with Nco I (P4+Nco I). PCR product corresponding to undigested SR-BI (476 bp), SR-BII (347 bp) and SR-BIII (541 bp) are indicated.

As an isoform of SR-BI, SR-BII, is expressed in mouse tissue and human cell lines, primers able to detect both isoforms were used (Figure 3A). Total RNA was isolated from macrophages from healthy volunteers and from atherosclerotic plaque tissue using the RNeasy kit (Qiagen, Hilden, Germany). RNA was reverse transcribed using random hexamers and 20 units AMV-reverse transcriptase (Promega Corp., Madison, WI). PCR was performed in Taq buffer (Roche Diagnostics, Mannheim, Germany) with 2 units of Taq polymerase (Roche Diagnostics), 1 μM of primers hCLA-1 z5 (5'-GGG AAG ATC GAG CCA GTA-3'; Genset, Paris, France) and hCLA-1 z3 Not I (5'-GCG CGG CCG CGG GGA CAG TGT GAC ATC T-3'; Genset), 400 ng cDNA and dNTPs (0.2 mM each) using GeneAmp PCR system 9600 (Perkin-Elmer, Foster City. CA). The amplification is predicted to generate a 476 bp fragment for the SR-BI transcript and a 347 bp fragment for the SR-BII transcript. PCR products and Nco I-digested PCR products were separated on a 3% agarose gel containing ethidium bromide. The PCR products were cloned into the pCRII vector (Invitrogen, San Diego, CA) and verified by DNA sequencing.

### Statistical analysis

Statistical analyses were performed using Student's *t*-test or by ANOVA followed by Student's *t*-test.

## Results

### Effect of hypoxia on macrophage SR-BI expression

As the thickness of the atherosclerotic plaque increases, diffusion of oxygen is decreased and zones of low oxygen tension can be detected within atherosclerotic plaques. Several studies have shown that hypoxia alters a large number of macrophage functions [2]. We therefore investigated if macrophage SR-BI expression is regulated by hypoxia *in vitro*. Human macrophages from 13 healthy volunteers were exposed to hypoxia for 24 h. The expression of macrophage SR-BI was decreased from 1.26 ± 0.17 arbitrary units to 0.73 ± 0.10 arbitrary units (mean ± SEM; p < 0.005) after hypoxia exposure as determined by real-time RT-PCR analysis.

### Effect of mmLDL on macrophage SR-BI expression

**Figure 1:**
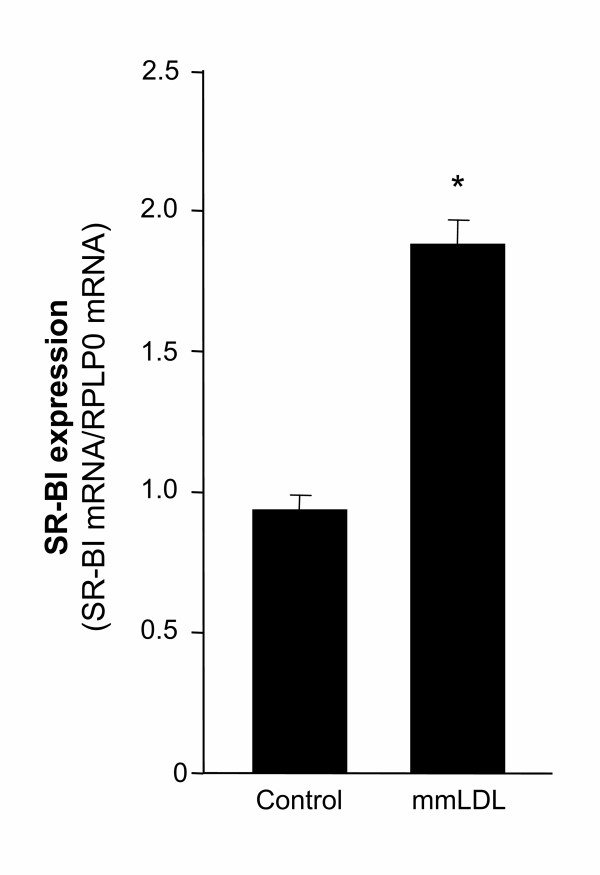
**Effect of mmLDL on the expression of SR-BI in macrophages**. Macrophages from 4 different healthy voluntary donors were exposed to mmLDL (50 μg protein/ml) for 24 h and SR-BI gene expression was analyzed by real-time RT-PCR using RNA from mmLDL treated and untreated control macrophages. The primers for real-time RT-PCR analysis used in this experiment were designed to detect SR-BI and the SR-BI isoforms. SR-BI expression was normalized to the reference gene RPLP0. The results are presented as mean +/- SEM. *, p < 0.005.

The uptake of lipids and cholesterol by macrophages is a key event implicated in the development of atherosclerosis. Therefore, scavenger receptor expression is thought to be a critical determinant of lipid accumulation in macrophages. Human macrophages from 4 healthy volunteers were treated with mmLDL (50 μg protein/ml) for 24 h. The expression of SR-BI was significantly up regulated in macrophages after mmLDL treatment compared to control macrophages (p < 0.05, Figure 1) as determined by real-time RT-PCR analysis.

### Expression of SR-BI in macrophages derived from subjects with atherosclerosis

**Figure 2:**
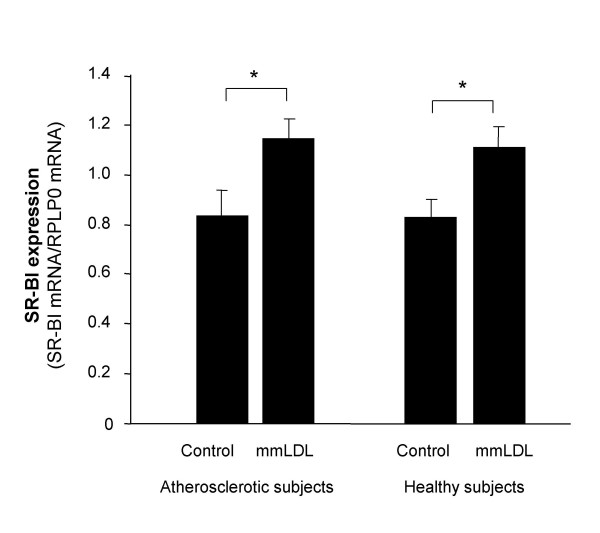
**SR-BI expression in macrophages from atherosclerotic subjects**. Macrophages from 15 atherosclerotic subjects and 15 healthy matched control subjects were treated with mmLDL (50 μg protein/ml) for 24 h. SR-BI expression was analyzed in both groups both before and after mmLDL treatment by real-time RT-PCR. SR-BI expression was normalized to the reference gene RPLP0. The primers for real-time RT-PCR analysis used in this experiment detect SR-BI and the SR-BI isoforms. The results are presented as mean +/- SEM. *, p < 0.05.

As SR-BI expression in macrophages may modulate the development of atherosclerosis we investigated if macrophages derived from subjects with atherosclerosis displayed an altered expression pattern compared to macrophages derived from matched healthy controls. SR-BI gene expression in macrophages and mmLDL-treated macrophages from 15 atherosclerotic subjects and 15 controls (the macrophage INTERGENE subjects) were analyzed by real-time RT-PCR (Figure 2). No difference in gene expression was detected between the subjects with atherosclerosis and the matched healthy controls (Figure 2). However, the analysis confirms the previous findings that SR-BI expression is increased in response to 24 h mmLDL treatment of macrophages from both the subjects with atherosclerosis and the control subjects (p < 0.05 and p < 0.05, respectively). Macrophage SR-BI expression levels were not correlated to serum HDL cholesterol levels in the macrophage INTERGENE subjects (data not shown).

### Analysis of SR-BI isoforms in macrophages and atherosclerotic plaques

**Figure 4:**
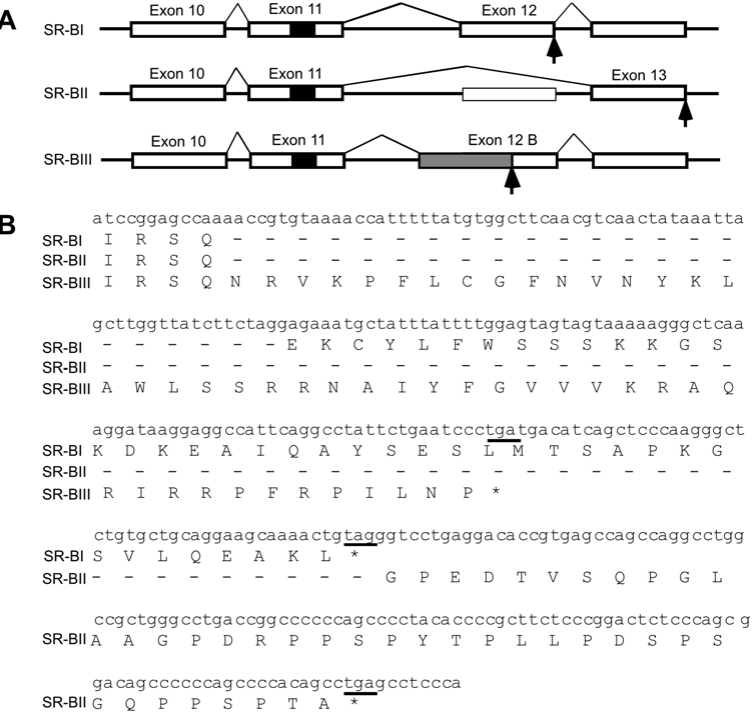
**Alternative splicing generating SR-BI, SR-BII, and SR-BIII transcripts**. Schematic illustration of the predicted alternative splicing generating the SR-BI, SR-BII, and SR-BIII transcripts (A). White boxes indicate exons, arrows indicate stop codons, black boxes indicate the part coding for the C-terminal transmembrane domain and the gray box indicates the alternative reading frame used in the SR-BIII isoform. Nucleotide and amino acid sequence of the human SR-BI, SR-BII, and SR-BII isoforms (B). Putative stop codons are underlined.

As different isoforms of SR-BI may modulate receptor function [23], the expression of SR-BI isoforms was investigated in human macrophages by RT-PCR using primers designed to detect both SR-BI and SR-BII transcripts. SR-BI (476 bp) and SR-BII (347 bp) transcripts were detected in all the samples examined (Figure 3B). The identities of the PCR products were verified by restriction enzyme mapping. Digestion with Nco I resulted in a 53-bp decrease in fragment size of both SR-BI (423 bp) and SR-BII (294 bp) as predicted from the mRNA sequence. Sequence analysis of the 347 bp PCR product (clones from 2 subjects) confirmed that this isoform is the human equivalent to the SR-BII isoform originally cloned in mouse. An additional PCR product that decreased in size with Nco I digestion was also detected. The PCR product (541 bp) was sequenced (clones from 2 subjects) and identified as a novel isoform of the SR-BI receptor and was therefore designated SR-BIII (GenBank accession number AF254409). This novel isoform appears to be generated by the use of an alternative splice acceptor site in intron 11, resulting in a frame shift after aa 467, and can be predicted to generate a unique intracellular C-terminal domain containing 53 aa (Figure 4A and 4B).

To analyze whether the SR-BI isoforms were also expressed in human atherosclerotic plaque tissue, the experiment was repeated using RNA samples from plaques obtained from four patients that underwent carotid endarterectomy. PCR products corresponding to SR-BI (476 bp), SR-BII (347 bp) and SR-BIII (541 bp) were detected in atherosclerotic plaques from all four patients analyzed (Figure 3C).

### Analysis of the intracellular C-terminal domain of the SR-BI isoforms

**Figure 5:**
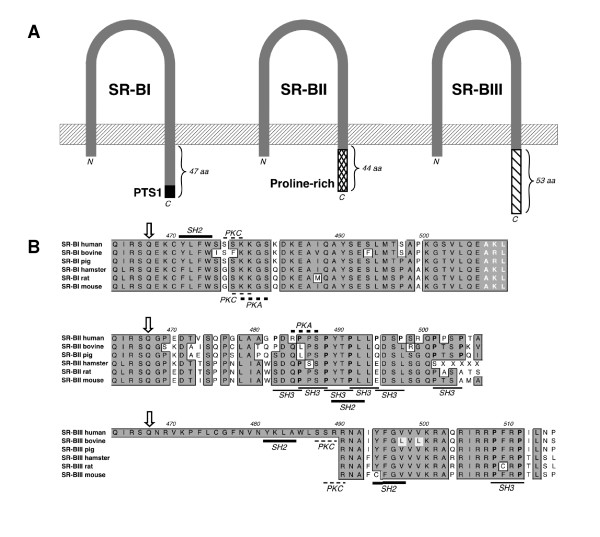
**Intracellular domains of SR-BI isoforms**. Schematic illustration of the topology of the three isoforms (A). The PTS1 domain of SR-BI, the proline-rich domain of SR-BII and the lengths of the intracellular domains are indicated. Species alignment of the intracellular domain of SR-BI, SR-BII and SR-BIII (B). Bovine, pig, hamster, rat and mouse SR-BIII sequences were deduced from SR-BI mRNAs. Sequence identities (dark gray boxes) are indicated. The isoforms differ in amino acid sequence downstream of Gln-467 (arrow). The PTS1 motif of SR-BI (white bold characters), and proline residues of the Src Homology (SH3) binding motif (black bold) are indicated. Potential sites for protein kinases A and C dependent phosphorylation, SH2 and SH3 binding motifs are indicated by bars.

SR-BI and SR-BI isoforms are predicted to contain two transmembrane domains and two intracellular domains at the amino (N)- and carboxy (C)-termini, respectively. The N-terminal intracellular domain of SR-BI is predicted to contain 8 amino acids (aa) and the C-terminal intracellular domain of SR-BI is predicted to consist of 47 aa. A peroxisomal targeting sequence (PTS1) is present in the C-terminal domain of SR-BI, which is not present in the SR-BII and SR-BIII isoforms [8,24]. The intracellular C-terminal domain of human SR-BII contains 44 aa and has a high content of proline aa (12 of 44 aa). The predicted amino acid sequences of SR-BII from other species also have high proline content in the intracellular C-terminal domain (Figure 5B). The prolines in human SR-BII are in some places interspersed with two other amino acids generating PXXP motifs. The PXXP motif is the minimal consensus binding-motif for src homology 3 (SH3) domain-containing proteins [25]. One of these PXXP motifs (aa 488–491 of SR-BII) is conserved between species (Figure 5B). The novel SR-BIII transcript is predicted to encode an intracellular C-terminal domain that is longer (53 aa) than the intracellular C-terminal domains of both SR-BI and SR-BII (Figure 5A). The C-terminal domain of SR-BIII is less proline-rich than SR-BII and one SH3 binding site in SR-BIII is conserved between species (Figure 5B).

## Discussion

The key role of SR-BI in the development of atherosclerosis has been shown in genetically modified mice models. In particular, hepatic overexpression of SR-BI reduces atherosclerotic lesion formation [9], which is attributed to the role of SR-BI in the uptake of HDL-cholesterol in the liver. This is supported by the finding that SR-BI disruption increases plaque formation [26]. Genetically modified mice models have also been used to investigate the role of macrophage SR-BI expression in the development of atherosclerosis. Bone marrow transplantation studies have shown that SR-BI disruption in macrophages augment the development of advanced atherosclerotic plaques [10,27]. This effect is attributed to SR-BI role in cholesterol efflux. However, a recent study by Van Eck *et al *shows that the development of early atherosclerotic plaque (fatty streaks) is reduced in macrophages lacking SR-BI expression [28]. This effect could be related to the scavenger receptor function of SR-BI by enhancing uptake of oxidized LDL or VLDL [28]. This indicates that macrophage SR-BI probably plays multiple roles in the development of atherosclerosis.

As the thickness of the atherosclerotic plaque increases, diffusion of oxygen is decreased and zones of low oxygen tension within atherosclerotic plaques have been detected [3]. In this study we show for the first time that SR-BI gene expression is down regulated by hypoxia. Reduced macrophage SR-BI gene expression during hypoxic condition may lead to reduced cholesterol efflux from macrophages in hypoxic zones of the atherosclerotic plaque and may increase foam cell formation in these areas.

Previous studies have shown disparate effect of highly oxidized LDL (oxLDL) on SR-BI expression in human monocyte-derived macrophages. Hirano *et al *reported an increased expression of SR-BI during macrophage differentiation and that oxLDL induced SR-BI mRNA and protein expression after 24 h of treatment [29]. In contrast, Han *et al *reported that 10 days differentiated macrophages displayed a decreased SR-BI expression in response to 16 h oxLDL treatment [30]. However, less differentiated macrophages (3 days differentiation) responded to 16 h of oxLDL treatment with increased SR-BI expression [30]. This indicates that the differentiation status of the macrophages and the time of oxLDL treatment influences SR-BI expression. In this study we show that SR-BI expression in macrophages is increased in response to 24 h of mmLDL treatment. Differences in chemical properties of mmLDL compared to oxLDL can affect macrophage SR-BI expression. It is unclear which of these *in vitro *foam cell formation models that best resembles foam cell formation *in vivo*.

It has been suggested that genetic variants involved in the development of complex disorders such as atherosclerosis, are often located in regulatory regions of the genome and affect the transcription of the susceptibility gene. As SR-BI gene expression in macrophages may modulate the development of atherosclerosis we investigated if macrophages derived from subjects with atherosclerosis displayed an altered expression pattern compared to macrophages derived from matched healthy controls. The results of this experiment indicate that altered expression of SR-BI in macrophages is not a common component in the development on atherosclerosis in the Swedish population. Recently, a variant of the human SR-BI promoter has been identified in a Taiwanese Chinese population. This promoter variant displays decreased gene expression and has been associated to increased plasma HDL cholesterol levels [16]. However, the allele frequency of this functional variant is rather low. SR-BI and members of the ATP-binding cassette transporters have been shown to facilitate cellular cholesterol efflux. SR-BI mainly binds phospholipid-rich HDL particles, whereas ABCA1 preferably uses lipid-poor HDL particles as cholesterol acceptors [31]. However, the precise role and interactions between these proteins is not completely understood.

The presence of multiple SR-BI isoforms in macrophages and atherosclerotic plaques makes the interpretation of the role of SR-BI in human atherosclerosis complex. We have previously identified a PTS1 motif in the C-terminus of SR-BI [8,24], which is not present in SR-BII and SR-BIII. The PTS1 motif is recognized by the peroxisomal targeting import receptor Pex5p [32], which mediates uptake of proteins in the peroxisome and may therefore be of importance for SR-BI action as the peroxisome is an important subcellular site for cholesterol and bile acid metabolism. A protein from rat liver membrane extracts named CLAMP has been shown to interact with the C-terminal end of SR-BI [33]. The human SR-BII isoform was also detected in macrophages and atherosclerotic plaques. The mouse SR-BII isoform binds HDL and mediates both cholesterol uptake and efflux but seems to be less efficient than SR-BI [23]. Lower surface expression or reduced stability of SR-BII compared to SR-BI may contribute to the differences between the two molecules [24]. It has recently been shown that SH3 domain containing proteins may interact with the SR-BII C-terminal domain [34]. This indicates that the different SR-BI isoforms may have distinct functional and signaling properties.

We have identified a novel isoform of SR-BI, SR-BIII, which is expressed in macrophages and atherosclerotic plaques. The SR-BIII isoform is probably generated by the use of an alternative splice acceptor site in intron 11 and encodes a unique intracellular C-terminal of the receptor. Further studies are needed to determine if SR-BIII also functions as a HDL receptor and what physiological function it may have.

## Conclusion

We conclude that SR-BI is regulated by proatherogenic stimuli such as hypoxia and mmLDL in human macrophages. However, no differences in SR-BI expression were detected between macrophages from subjects with atherosclerosis and healthy controls. This indicates that altered SR-BI expression is not a common feature of atherosclerosis. In addition, we identified SR-BIII as a novel SR-BI isoform expressed in human macrophages and in human atherosclerotic plaques.

## Competing interests

The author(s) declare that they have no competing interests.

## Authors' contributions

All the authors have contributed to the design of the study, the data analysis and the writing of the manuscript. P-A.S. performed RT-PCR analysis and bioinformatical analysis. M.C.O.E. and B.O. performed the macrophage culture and mmLDL treatment. M.S.C.S carried out the sequencing and sequence analysis. D.H: performed the real-time RT-PCR analysis. V.S. collected the atherosclerosis plaque samples and extracted RNA. B.F. and D.T. coordinated and phenotyped the macrophage INTERGENE subjects, L.M.H. and O.W. contributed with the macrophage hypoxia experiments. L.M.S.C and B.C. designed the study, and participated in its coordination and data interpretation. The final version of the manuscript has been read and approved by all the authors.

## Pre-publication history

The pre-publication history for this paper can be accessed here:


